# Two-colour serial femtosecond crystallography dataset from gadoteridol-derivatized lysozyme for MAD phasing

**DOI:** 10.1038/sdata.2017.188

**Published:** 2017-12-12

**Authors:** Alexander Gorel, Koji Motomura, Hironobu Fukuzawa, R. Bruce Doak, Marie Luise Grünbein, Mario Hilpert, Ichiro Inoue, Marco Kloos, Gabriela Nass Kovács, Eriko Nango, Karol Nass, Christopher M Roome, Robert L Shoeman, Rie Tanaka, Kensuke Tono, Lutz Foucar, Yasumasa Joti, Makina Yabashi, So Iwata, Kiyoshi Ueda, Thomas R. M Barends, Ilme Schlichting

**Affiliations:** 1 Max-Planck-Institut für medizinische Forschung, Jahnstrasse 29, Heidelberg 69120, Germany; 2 Institute of Multidisciplinary Research for Advanced Materials, Tohoku University, Sendai 980-8577, Japan; 3 RIKEN SPring-8 Center, Kouto 1-1-1, Sayo, Hyogo 679-5148, Japan; 4 Department of Cell Biology, Graduate School of Medicine, Kyoto University, Yoshidakonoe-cho, Sakyo-ku, Kyoto 606-8501, Japan; 5 Japan Synchrotron Radiation Research Institute, Kouto 1-1-1, Sayo-cho, Sayo-gun, Hyogo 679-5198, Japan

**Keywords:** Molecular biophysics, Nanocrystallography

## Abstract

We provide a detailed description of a gadoteridol-derivatized lysozyme (gadolinium lysozyme) two-colour serial femtosecond crystallography (SFX) dataset for multiple wavelength anomalous dispersion (MAD) structure determination. The data was collected at the Spring-8 Angstrom Compact free-electron LAser (SACLA) facility using a two-colour double-pulse beam to record two diffraction patterns simultaneously in one diffraction image. Gadolinium lysozyme was chosen as a well-established model system that has a very strong anomalous signal. Diffraction patterns from gadolinium lysozyme microcrystals were recorded to a resolution of 1.9 Å in both colours. This dataset is publicly available through the Coherent X-ray Imaging Data Bank (CXIDB) as a resource for algorithm development.

## Background & Summary

SACLA is one of the two currently operating X-ray free-electron laser (XFEL) facilities in the world (with several XFELs coming online this year) producing a hard X-ray beam for the investigation of matter, with various applications in biology^1,2^, chemistry, physics and material science. In general, XFELs have the capacity to deliver a pulsed beam that is ten billion times brighter than synchrotron X-ray beams, with pulses ten thousand times shorter. So far, single wavelength anomalous dispersion (SAD)^3–10^ and single or multiple isomorphous replacement (SIR^10,11^, MIR^12^) methods have been used for *de novo* structure determination with XFELs to solve the phase problem. However, the stochastic nature of the experiment (randomly oriented crystals, strongly varying pulse intensity and wavelength distribution) demands large amounts of sample and beam time to average out the stochastic effects. Since 2013, two distinct X-ray pulses^13^ that have an unprecedentedly large energy separation can be created simultaneously by the split undulator operation of the SACLA XFEL. Thus, a large and flexible wavelength separation of the double-pulse of more than 30% with a precisely controlled time interval down to the attosecond regime can be realized. This allows targeting of multiple absorption edges of a suitable anomalous scatterer simultaneously. Using this operation mode of the SACLA XFEL, two distinct datasets can be recorded from the same sample thereby reducing the costs in both beam time and sample for structure determination significantly.

Here we describe the deposition of a two-colour serial femtosecond crystallography (SFX) dataset acquired at SACLA as reported in Gorel *et al.*^14^. The two diffraction patterns were first identified and then processed individually for successful MAD phasing. Analysis of this dataset showed that 5,000 images were required for MAD-based *de novo* structure determination. Furthermore, it was shown that phases retrieved by MAD phasing are more accurate than phases retrieved by SAD phasing using the same number of diffraction images.

## Methods

### Data acquisition

The two-colour experiment (proposal number 2015B8045) was performed in January 2016 at the SACLA XFEL in Hyogo, Japan. The photon energies for the two colours were chosen to be above the M-edges (7 keV) and L-edges (9 keV) of gadolinium, respectively. A High Viscosity Extrusion injector^15^ (HVE) mounted in the DAPHNIS^16^ chamber was used to introduce gadolinium lysozyme microcrystals^14^ suspended in grease medium^17^ into the two-colour double-pulsed X-ray beam. X-ray diffraction data was recorded at beamline 3 at the SACLA facility using the multiport CCD (MPCCD^18^) detector. Two diffraction patterns, one for each colour, were acquired simultaneously in one diffraction image.

### Data analysis

Data analysis was performed on the SACLA High Performance Computing Cluster consisting of several steps of parameter optimization and special data processing for the two-colour data. In the following we provide a more detailed description of the work published in Gorel *et al.*^14^.

### Raw data hit files

During a period of 12 h, a dataset of 570,000 diffraction images with consistent experimental parameters (attenuation, transmission, detector distance etc.) was collected. From this set 208,373 hits were identified with the programme Cheetah^19^ (40.6% of the total dataset). The raw data images were obtained using the Cheetah Dispatcher^20^ graphical user interface (GUI) from the set of all recorded images as a subset of images with more than 20 peaks. By default, this data contains only the wavelength of the 7 keV colour pulse saved in the HDF5 dataset 'photon_energy_ev' and 'photon_wavelength_A' in the diffraction image. The raw images were written in the multi-event data format and thus had to be split into individual diffraction images using the split.py module before further data processing was performed.

### Silicon powder files

Silicon powder patterns were recorded and used for detector distance parameter determination. To this end, grease was mixed with silicon nanocrystals and attached to the injector capillary tip. To calculate the detector distance, the radii of the Debye Scherrer diffraction rings had to be retrieved. However, due to the low concentration of silicon nanocrystals the high resolution powder rings were faint and could not be distinguished from the background by a threshold approach. This made another pattern recognition technique necessary. Thus, 300 interest points^14^ (i.e., points with certain characteristics like signal over background and signal divided by background exceeding a dynamically calculated value) per CCD (2,400 in total) were calculated for each diffraction image of the silicon powder data and written into the HDF5 dataset 'poi' in the diffraction image container. Using these interest points the detector distance was calculated to be 51.03 mm as described in the supplementary information of Gorel *et al.*^14^.

### Spectra files

By default, the raw-images in HDF5 file format contain only the wavelength value for one particular colour because the energy separation of the two colours exceeds the spectral range of the narrow range inline spectrometer. Thus, only the 7 keV colour or the 9 keV colour wavelength is available for each shot but not both at the same time. Since knowledge of the wavelength values of both colours is required for the two-colour data processing, the missing wavelength had to be retrieved from the recordings of a wide range inline spectrometer^21^. To this end, the measured spectral profiles were recorded into spectra.h5 files for later use by the write_calib_color.py module to obtain accurate wavelength values and to add these to the diffraction images. The wide range inline spectrometer images were obtained with the application programming interface to SACLA metadata database. Spectral profiles were obtained from these 1,024×512 pixel images by collapsing them into a 1,024 pixel sized one-dimensional image. These profiles were recorded into a HDF5 dataset named 'spectrum' within the spectra.h5 files by the write_spectra.py module. A double Lorentzian model was fitted to these profiles resulting in values for the amplitudes of both peaks, a constant offset, the positions of both Lorentzians and their widths. These parameters were written into the HDF5 datasets 'Amp1', 'Amp2', 'Const', 'Peak1', 'Peak2', 'Width1' and 'Width2', respectively, in the spectra.h5 file by the write_spectra.py module. The HDF5 dataset 'tags' in the spectra.h5 file contains the tag name of the respective diffraction image and thus identifies the set of parameters of the two-colour double-pulse that belongs to the respective diffraction event. Two calibration runs are available (run 392,732 for 7 keV and run 392,738 for 9 keV colour) which were used to find the photon energy calibration functions for the respective energy ranges of the wide range inline spectrometer.

Since the resolving power of the narrow range spectrometer is smaller than that of the wide range spectrometer, the same energy value is retrieved for different readings of the wide range spectrometer. By calculating the median values from the wide range spectrometer readings that have the same narrow range spectrometer reading, calibration points were obtained, which were then used for the photon energy calibration function estimation as described in Gorel *et al.*^14^ supplement.

### 7 keV and 9 keV indexable files

The respective photon energy calibration function was applied by the write_calib_color.py module to the double-pulse energy profile fit parameters. This way the photon energy was obtained and written into the HDF5 datasets 'photon_energy_ev_color1', 'photon_energy_ev_color2', 'photon_wavelength_A_color1' and 'photon_wavelength_A_color2', where color1 corresponds to the 7 keV and color2 to 9 keV colour. The raw data was processed by CrystFEL's^22^ indexamajig with the colour information added, against given cell parameters (*a*=*b*=78.3 Å, *c*=39.1 Å α=β=γ=90°). All correctly indexed images are contained in the hits7kev (21,830) and hits9kev (33,297) datasets.

### Two-colour indexable files

**Figure 1:**
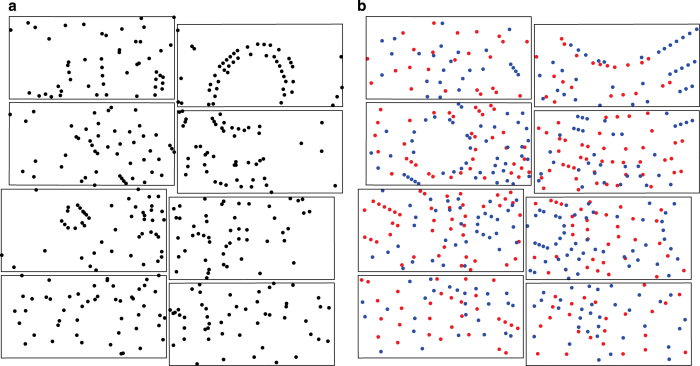
Diffraction Patterns. (**a**) The two similar diffraction patterns in this image likely belong to a twinned crystal. Indexing in a second colour was not possible. (**b**) The diffraction patterns from 7 keV (red) and 9 keV (blue) strongly differ since very different areas in reciprocal space are probed by the two-colour double-pulse.

Due to an anti-correlation of the double-pulse intensities and thus of the respective diffraction pattern intensities, the two-colour images typically contain one strong and one weak diffraction pattern. Because two completely different diffraction patterns are present in the diffraction image (see Fig. 1) and since the processing programme can deal with only one pattern at a time, processing of the two-colour diffraction data is not straightforward and was achieved by the following three steps: Firstly, the diffraction images were processed with CrystFEL's indexamajig module for the brighter colour identifying the stronger diffraction pattern in the diffraction image (threshold 200, signal-to-noise ratio 5). Secondly, before the other diffraction pattern of the weaker colour could be processed, the peak search parameters, i.e., threshold and signal-to-noise ratio, were lowered to select a broader set of diffraction peaks by thresholding (threshold 150, signal-to-noise ratio 3). Then, all peaks of the stronger diffraction pattern were removed with the write_subtract.py module from this set such that the residual peaks possibly constituted the weaker diffraction pattern. Subsequently, in the third step these peaks were processed with indexamajig. To compensate for the residual error in peak position prediction, large integration radii were applied with indexamajig (--int-radius=6,6,8).

For two-colour indexing all 9 keV indexable images from the hits9kev dataset were reprocessed to identify the second (weak) diffraction pattern. These points were saved in the HDF5 dataset 'residual_points_7keV' in the diffraction images and were processed with CrystFEL’s indexamajig module. Thus, 14,782 two-colour indexable images were found from the 33,297 images of the hits9kev data subset (44.4%). An overview of all HDF5 data fields used in data processing is provided in Table 1. A summary of all parameters used in data processing is given in Table 2 as well as an overview of the indexing rates at the various stages of data analysis in Table 3.

### Mean phase error

A reference structure was built using data from all 14,782 9 keV indexable images. The cosine difference defined as Cos[phase(model_obtained_with_all_images)]-Cos[phase(model_obtained_with_subset_of_images)] was calculated to assess the quality of the phases. This is a comparison between a well-defined reference structure and the structure obtained with fewer images. By contrast, the figure of merit is an intrinsic measure without reference. The calculated figure of merit and the cosine differences are tabulated in Table 4 along with the number of correctly built residues by ARP/wARP.

### Code availability

For data processing CrystFEL 0.6.2 and newly implemented python modules were employed. CrystFEL 0.6.2 is a free open source software under the GNU Public License version 3 and can be downloaded from http://www.desy.de/~twhite/crystfel/. stream2h5.py, write_calib_color.py, write_pca_peaks.py, write_spectra.py, split.py and write_subtract_peaks.py are free open source software under the GNU Public License version 3 and can be downloaded from https://github.com/AlexanderGorel/crystallography.

## Data Records

Due to the large size of the raw data we only deposited the hit images (hitsrawdata.tar) in the Coherent X-ray Imaging Data Bank website (CXIDB) with the CXIDB ID 66 (Data Citation 1). Furthermore, we deposited the spectra.h5 files (spectra.tar.gz) with spectrum information, the silicon powder files (silicon-powder-poi.tar.gz) with the calculated interest points, the 7 and 9 keV indexable data (hits7kev.tar and hits9kev.tar) and the two-colour indexable data (two-color.tar) with residual points from the weak diffraction pattern in HDF5 file format as well as the geometry file and the cell file (supplement-files.tar.gz) at CXIDB (Data Citation 1).

## Technical Validation

We have successfully phased the deposited data by MAD and solved the structure of the lysozyme gadoteridol complex using AutoSHARP^23^ with data to 1.9 Å resolution. The final structure built by ARP/wARP^24^ was refined against 5,000 9 keV diffraction images. It is available from the Protein Data Bank (Data Citation 2).

## Usage Notes

Our complete data processing scheme is shown in the data flow diagram published in Gorel *et al.*^14^ supplement. Each data processing step can be repeated with the provided command line tools. It would be interesting to see a comparison between our processing strategy and others, such as the recently published FELIX^25^ algorithm which is capable of indexing more than ten different diffraction patterns per diffraction image. Moreover, the two-colour data set can be used for further software development. Indexing the reflections belonging to one colour yields the orientation matrix of the unit cell relative to the laboratory system. Future software may then use this matrix as a starting point for the initial indexing of the Bragg reflections of the second colour. Since they provide a different set of diffraction conditions, the matrix can be optimized for the second colour and through iterative refinement using the two sets of reflections, an extremely accurate orientation matrix can be obtained, in particular for the weak high resolution reflections. Ideally, a global refinement including both colours should be performed. We expect that such new analysis algorithms will greatly improve serial femtosecond crystallography (SFX) data processing in general and facilitate MAD phasing at XFELs in particular.

## Additional information

**How to cite this article:** Gorel, A. *et al.* Two-colour serial femtosecond crystallography dataset from gadoteridol-derivatized lysozyme for MAD phasing. *Sci. Data* 4:170188 doi: 10.1038/sdata.2017.188 (2017).

**Publisher’s note:** Springer Nature remains neutral with regard to jurisdictional claims in published maps and institutional affiliations.

## Supplementary Material



## Figures and Tables

**Table 1:** Overview of the data fields.

**Dataset Name**	**HDF5 Data Field Name**	**Data Type**
silicon-powder-poi	data	Dataset {8192, 512}
	photon_energy_ev	Dataset {SCALAR}
	photon_wavelength_A	Dataset {SCALAR}
	poi	Dataset {2400, 3}
Spectra	Amp1	Dataset {5150}
	Amp2	Dataset {5150}
	Const	Dataset {5150}
	Peak1	Dataset {5150}
	Peak2	Dataset {5150}
	Width1	Dataset {5150}
	Width2	Dataset {5150}
	spectrum	Dataset {5150,1024}
	tags	Dataset {5150}
Hitsrawdata	data	Dataset {8192, 512}
	photon_energy_ev	Dataset {SCALAR}
	photon_wavelength_A	Dataset {SCALAR}
hits7kev	data	Dataset {8192, 512}
hits9kev	pca_peaks	Group
two-color	pca_peaks/100	Dataset {800, 3}
	photon_energy_ev_color1	Dataset {SCALAR}
	photon_energy_ev_color2	Dataset {SCALAR}
	photon_wavelength_A_color1	Dataset {SCALAR}
	photon_wavelength_A_color2	Dataset {SCALAR}
	residual_points_7keV	Dataset {n, 3}
	residual_points_9keV	Dataset {n, 3}
(silicon-powder-poi) ‘data’ contains the diffraction image of the Debye Scherrer powder ring patterns while ‘poi’ contains the calculated interest points, 300 per CCD. (spectra) ‘Amp1’, ‘Amp2’, ‘Const’, ‘Peak1’, ‘Peak2’ contain the fit parameters for the double-Lorentzian beam energy profile; ‘spectrum’ contains the energy profile as a 1,024 pixel image. ‘tags’ contains the name of the respective diffraction image. (hitsrawdata) ‘data’ contains the diffraction image. (hits7kev, hits9kev, two-color) ‘data’ contains the diffraction image. ‘pca_peaks/100’ contains the calculated interest points, 100 per CCD. ‘photon_energy_ev_color1’ contains the photon energy of the 7 keV colour while ‘photon_energy_ev_color2’ contains the photon energy of the 9 keV colour. The weak n diffraction pattern points are saved in ‘residual_points_7keV’ or ‘residual_points_9keV’, respectively if they belong to the 7 keV or the 9 keV colour.		

**Table 2:** Summary of the processing parameters.

Detector distance	51.03 mm
Calibration function 7 keV colour	(6.58984+0.00298 x) keV
Calibration function 9 keV colour	(5.72503+0.00381 x) keV
Cell parameters	a=b=78.3 Å, c=39.1 Å, α=β=γ=90°
Processing parameters for strong diffraction pattern	threshold 200, signal-to-noise ratio 5
Processing parameters for weak diffraction pattern	threshold 150, signal-to-noise ratio 3
Integration radii parameters	6,6,8
The detector distance was optimized using the Debye Scherrer ring diffraction patterns from silicon nanopowder. The calibration functions for the wide range inline spectrometer were obtained from calibration runs. The x argument is for the position (in pixels) of the Lorentzians in the double pulse energy profile image to obtain the precise energies of the two colours (7 and 9 keV). Processing parameters were varied to obtain the strong and the weak diffraction patterns. Large integration parameters were chosen to compensate the prediction uncertainty of the diffraction patterns due to residual uncertainties in wavelength and detector distance.	

**Table 3:** Indexing rate of the 208,373 hits at the various stages of the analysis.

**Processing step**	**Number of indexed images**		
	**7 keV**	**9 keV**	**7 and 9 keV**
**No optimization**	8,322 (4%)	10,374 (5%)	684 (0.3%)
**Distance, wavelengths optimized**	21,830 (10.5%)	33,297 (16.0%)	2,129 (1%)
**Peaks of dominant pattern removed from search list**	21,830 (10.5%)	33,297 (16.0%)	23,144 (11.1%)

**Table 4:** Final phasing statistics.

**No. of images**	**Phasing Method**	FOM*	**No. residues in first round (sequenced)**	**No. residues in second round (sequenced)**	**Mean Cosine Difference**
9,000	MAD	0.529	127 (115)	127 (127)	0.372
	SAD	0.511	125 (104)	127 (127)	0.744
6,000	MAD	0.493	123 (112)	126 (126)	0.398
	SAD	0.475	124 (95)	124 (124)	0.753
5,000	MAD	0.473	115 (81)	127(127)	0.435
	SAD	0.457	49 (0)	120 (120)	0.759
Comparison of SAD phasing using only 9 keV data and MAD phasing using 9 and 7 keV data.					

*FOM: figure of merit: cosine of the phase error as estimated by AutoSHARP.
